# Limited carbon cycling due to high-pressure effects on the deep-sea microbiome

**DOI:** 10.1038/s41561-022-01081-3

**Published:** 2022-11-28

**Authors:** Chie Amano, Zihao Zhao, Eva Sintes, Thomas Reinthaler, Julia Stefanschitz, Murat Kisadur, Motoo Utsumi, Gerhard J. Herndl

**Affiliations:** 1 Department of Functional and Evolutionary Ecology, Bio-Oceanography and Marine Biology Unit, University of Vienna, Vienna, Austria; 2 Instituto Español de Oceanografía-CSIC, Centro Oceanográfico de Baleares, Palma de Mallorca, Spain; 3 Faculty of Life and Environmental Sciences, University of Tsukuba, Tsukuba, Ibaraki Japan; 4 Microbiology Research Center for Sustainability, University of Tsukuba, Tsukuba, Ibaraki Japan; 5 NIOZ, Department of Marine Microbiology and Biogeochemistry, Royal Netherlands Institute for Sea Research, Utrecht University, Texel, The Netherlands; 6 Vienna Metabolomics & Proteomics Center, University of Vienna, Vienna, Austria; 7 Present Address: Marine Evolutionary Ecology, Deep-Sea Biology Group, GEOMAR Helmholtz Centre for Ocean Research Kiel, Kiel, Germany

**Keywords:** Carbon cycle, Microbial biooceanography, Microbial ecology

## Abstract

Deep-sea microbial communities are exposed to high-pressure conditions, which has a variable impact on prokaryotes depending on whether they are piezophilic (that is, pressure-loving), piezotolerant or piezosensitive. While it has been suggested that elevated pressures lead to higher community-level metabolic rates, the response of these deep-sea microbial communities to the high-pressure conditions of the deep sea is poorly understood. Based on microbial activity measurements in the major oceanic basins using an in situ microbial incubator, we show that the bulk heterotrophic activity of prokaryotic communities becomes increasingly inhibited at higher hydrostatic pressure. At 4,000 m depth, the bulk heterotrophic prokaryotic activity under in situ hydrostatic pressure was about one-third of that measured in the same community at atmospheric pressure conditions. In the bathypelagic zone—between 1,000 and 4,000 m depth—~85% of the prokaryotic community was piezotolerant and ~5% of the prokaryotic community was piezophilic. Despite piezosensitive-like prokaryotes comprising only ~10% (mainly members of Bacteroidetes, *Alteromonas*) of the deep-sea prokaryotic community, the more than 100-fold metabolic activity increase of these piezosensitive prokaryotes upon depressurization leads to high apparent bulk metabolic activity. Overall, the heterotrophic prokaryotic activity in the deep sea is likely to be substantially lower than hitherto assumed, with major impacts on the oceanic carbon cycling.

## Abstract (web summary)

In situ measurements reveal that high pressure in the deep ocean doesn’t lead to elevated community-level microbial metabolic rates, in contrast to previous shipboard analyses made at atmospheric pressure.

## Main

The water column of the deep sea is a dark and typically cold realm (0–4 °C) with hydrostatic pressure increasing with depth. Prokaryotic abundance and activity decrease with depth, generally interpreted as a reflection of decreasing energy supply rates with depth^1^. After the submersible *Alvin* accidently sank almost 50 years ago, a previous study found that food left in *Alvin* at 1,540 m depth for more than 10 months was remarkably well-preserved^2^. They concluded that the high hydrostatic pressure prevented deep-sea microbes from utilizing this food source. Subsequently, studies on the effect of hydrostatic pressure on deep-sea prokaryotes were performed^3^; however, they revealed inconclusive results. Contrasting results on the impact of high pressure on deep-sea microbial communities might be due to differences in substrate concentrations used to determine metabolic rates and/or variable physical conditions of the water column such as down- or upwelling, or high temperatures of deep waters such as those characteristic for the Mediterranean Sea (~13 °C), which can influence the metabolism and physiology of deep-sea microbes^2–6^. Owing to the methodological difficulties in measuring prokaryotic activity under in situ pressure conditions, only a few comparative measurements of prokaryotic activity under in situ pressure and depressurized conditions are available from the meso- and bathypelagic global ocean, despite the potential impact hydrostatic pressure might have on deep-sea microbial activity and on understanding the ocean biogeochemical cycle^3,7–9^.

While the activity of sea-surface microbial communities is reduced or inhibited by hydrostatic pressure at about 10 MPa (corresponding to a depth of 1,000 m)^10^, some deep-sea microbes exhibit a piezophilic (that is, optimal growth at pressures >0.1 MPa) and piezotolerant lifestyle with specific adaptions to high hydrostatic pressure, low temperature and low nutrient conditions^11^. Comparing genomes from obligate piezophilic and piezosensitive microbes grown under low temperature (optimal growth of the piezophiles at 6–10 °C) indicated an adaptation to high hydrostatic pressure in piezophiles in membrane fluidity, stress response and cell motility^12^, consistent with previous culture-based studies^11^.

Commonly, the heterotrophic prokaryotic carbon demand (PCD) of deep-sea microbes is calculated from heterotrophic biomass production and respiration measurements based on shipboard incubations under atmospheric pressure conditions, assuming that pressure changes do not affect metabolic rates. Estimates of the PCD in the meso- and bathypelagic layers of the Atlantic revealed that the PCD is about one order of magnitude higher than the supply of particulate organic carbon (POC) via sinking particles^13^. A similar conclusion was reached for the Pacific albeit using a different approach^14^. This mismatch between the PCD and POC supply via sinking particles indicates some fundamental errors in our estimates on deep-sea prokaryotic activity and/or on the magnitude of sinking organic matter flux^1,9,15,16^.

## Heterotrophic microbial activity at in situ pressure conditions

**Extended Data Fig. 1:**
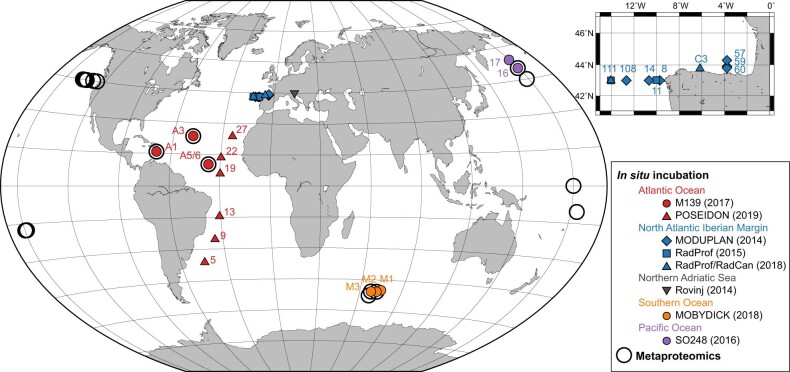
Sampling location of stations where the in situ microbial incubator (ISMI) was deployed and metaproteomic analyses were performed. The ISMI was deployed during the M139 and POSEIDON cruise in the Atlantic Ocean, MODUPLAN, RadProf and RadCan cruises in the North Atlantic off the Iberian Peninsula, MOBYDICK cruise in the Southern Ocean, and SO248 cruise in the Pacific Ocean, and at the Ruđer Bošković Institute, Rovinj, Croatia. Numbers indicate station names. Numbers in brackets indicate the year when sampling was performed. The coordinates of the stations are indicated in Supplementary Table 1. Detailed information of the proteomics stations can be found elsewhere^28^. The map was generated by The Generic Mapping Tools.

**Extended Data Fig. 2:**
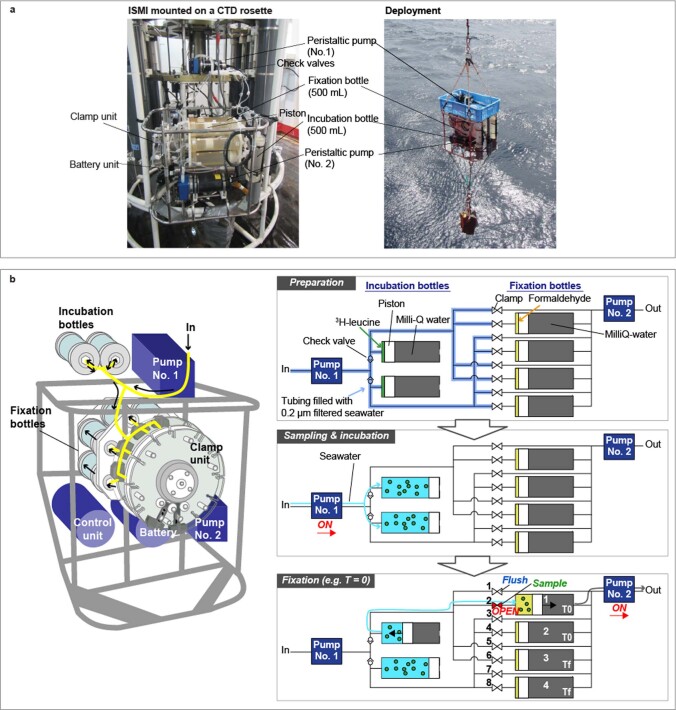
Overview of the ISMI. **a**, The ISMI can be mounted on a rosette sampling system or lowered by the shipboard winch. **b**, Schematic overview of the ISMI. There is only one inlet (left side of the figure) and one outlet (right side) in the system. Prior to deployment, the substrate and the fixative reagent are added into the incubation and fixation cylindrical sampler, respectively. All tubes are pre-filled with either 0.2 µm filtered seawater or MilliQ water. Cylindrical samplers from No. 1 to 4 collect samples in this order by opening the clamps from No. 1 to 8. There is always a flushing step prior to the actual sampling. Incubations are performed either in duplicate or in triplicate.

The heterotrophic prokaryotic activity was determined under in situ pressure conditions throughout the water column down to bathypelagic layers in the Atlantic, Pacific and Indian sector of the Southern Ocean (Extended Data Fig. 1 and Supplementary Table 1). Heterotrophic prokaryotic activity was assessed via the incorporation of radiolabelled leucine into proteins^17^ using an in situ microbial incubator (ISMI; Extended Data Fig. 2). The ISMI collects and incubates water at depths down to 4,000 m with substrate added such as ^3^H-leucine at the depth of sampling. Thus, the ISMI allows determination of prokaryotic activity without changes of the hydrostatic pressure and temperature, hence under in situ conditions (see Methods). The results obtained from these in situ incubations using the ISMI were compared with measurements on samples collected at the same site and depth as those of ISMI but under atmospheric pressure onboard the respective research vessel. Care was taken to prevent any contamination with organic and inorganic matter in all incubation bottles, and the incubation temperature was the same as the temperature in the in situ incubations (Methods and Supplementary Table 2).

**Fig. 1:**
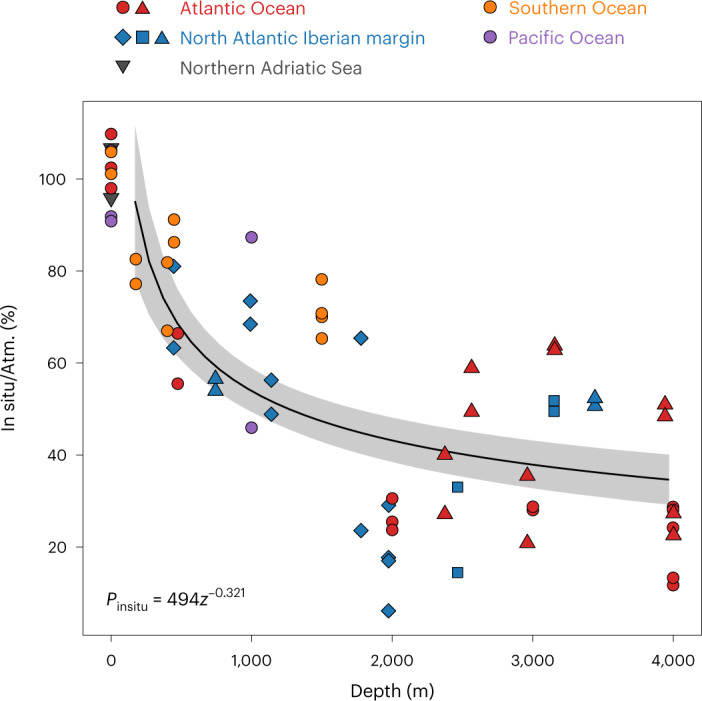
In situ bulk leucine incorporation rates normalized to rates obtained at atmospheric pressure conditions. Symbols correspond to the different research expeditions (Extended Data Fig. 1). Regression equation is a power law function: *P*_insitu_ = 494*z*^−0.321^ (*n* = 56, number of samples incubated at in situ), where *P*_insitu_ is the percentage of in situ leucine incorporation rate normalized to mean leucine incorporation rate under atmospheric pressure (Atm.) and *z* is depth (m). Shaded area indicates 95% confidence interval for the regression. Note that the data points at 0 m (*n* = 4) correspond to instrumental tests in which epi- to bathypelagic waters were incubated with the ISMI under atmospheric pressure conditions and compared with bottle incubations used for atmospheric pressure incubations to assess the potential bias associated with the instrument. These points are excluded from calculating the regression line. Source data

**Extended Data Fig. 3:**
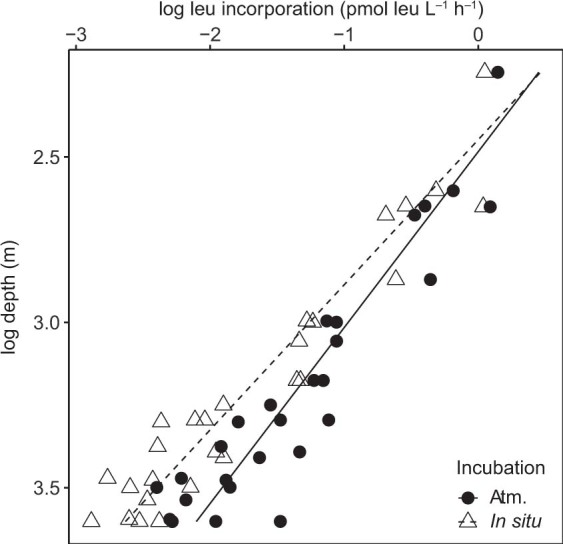
Vertical distribution of leucine incorporation rates incubated under in situ and atmospheric pressure conditions. Regressions: log (leucine incorporation) (pmol L^–1^ h^–1^) = –1.9*z* + 4.7 (atm.; *n* = 27, *r*^2^ = 0.87, *P* = 9.9 × 10^–13^); –2.3*z* + 5.6 (in situ; *n* = 27, *r*^2^ = 0.92, *P* = 3.9 × 10^–15^) where *z* is log depth in m. Sample size (*n*) indicates number of sites and depths (see Supplementary Table 1).

Generally, heterotrophic prokaryotic activity decreased with depth; however, under in situ pressure more than under atmospheric pressure conditions (analysis of covariance (ANCOVA) type III, *F* = 4.10, *P* = 0.048 for the slopes of log–log fits assuming power law function; Extended Data Fig. 3). For samples collected at 500 m depth, the impact of hydrostatic pressure was small, reaching about 75 ± 10% (mean ± s.d., *n* = 4) of the activity measured at atmospheric pressure (Fig. 1 and Supplementary Table 2). The difference in prokaryotic activity between in situ and atmospheric pressure conditions was most pronounced in the bathypelagic waters. In situ prokaryotic activity at ~1,000 m depth was ~60 ± 10% (mean ± s.d., *n* = 3) of that under atmospheric pressure. At the base of the bathypelagic waters (~4,000 m depth), in situ prokaryotic activity was only ~30 ± 15% (mean ± s.d., *n* = 4) of that measured under atmospheric pressure (Fig. 1 and Supplementary Table 2). Thus, bulk heterotrophic prokaryotic activity is greatly reduced in the bathypelagic realm under in situ pressure conditions. The question of whether most of the members of the microbial community are suppressed in their metabolic activity or only a small fraction respond to depressurization with elevated activity was addressed using single-cell activity measurements.

## Leucine incorporation rates at a single-cell level

**Fig. 2:**
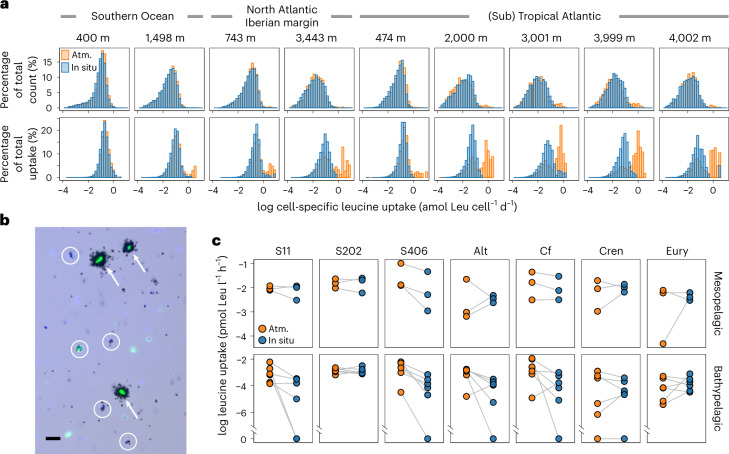
Cell-specific leucine uptake by prokaryotes. **a**, Distribution of cell-specific leucine uptake expressed as the percentage of total active cell counts (upper panels) and the percentage of total uptake (lower panels). Water was collected at meso- and bathypelagic depths and incubated under in situ and atmospheric pressure (Atm.) conditions (Supplementary Tables 1 and 2). **b**, A microscopic view of a bathypelagic sample (2,000 m) collected in the Atlantic and incubated under atmospheric pressure conditions. Black halos around the cells are silver grains corresponding to their activities. The highly active cells (>0.5 amol Leu cell^−^^1^ d^−^^1^, indicated by arrows) were barely found in in situ pressure incubations. Typical low-activity cells in the bathypelagic depths are indicated by circles. Green fluorescence, EUB338 probe mix; light blue, DAPI-stained cells. Scale bar, 5 µm. **c**, Leucine uptake by taxonomical groups: S11, SAR11 clade; S202, SAR202 clade; S406, SAR406 clade; Alt, *Alteromonas*; Cf, Bacteroidetes; Cren, Thaumarchaeota; Eury, Euryarchaeota. The grey line connects the same location and depth between in situ and Atm. samples representing the change in leucine uptake beween the two incubation conditions. Source data

Single-cell prokaryotic activity under in situ and atmospheric pressure conditions was determined on three mesopelagic (~400–750 m depth) and six bathypelagic samples (~1,500–4,000 m depth) collected in the Atlantic and Southern Ocean using microautoradiography with ^3^H labelled leucine combined with catalysed reporter deposition fluorescence in situ hybridization (Methods). Using microautoradiography, the silver grain halo around single cells indicating uptake of radiolabelled leucine serves as a proxy for single-cell prokaryotic activity^18,19^ (Methods). There was no detectable difference between in situ and onboard incubations at atmospheric pressure conditions in prokaryotic abundance (paired *t*-test, *P* = 0.724; Extended Data Table 1) and in the abundance of cells taking up leucine (paired *t*-test, *P* = 0.905). However, the total size of the silver grain halo around the cells taking up leucine, that is, cell-specfic leucine uptake, was higher under atmospheric pressure than under in situ hydrostatic pressure conditions (Extended Data Table 1). This is in agreement with the higher bulk leucine incorporation rates obtained under atmospheric than under in situ pressure conditions (Supplementary Table 2). Highly active cells (>0.5 amol leucine uptake cell^−^^1^ day^−^^1^) were found in the samples incubated under atmospheric pressure, hence depressurized conditions (Fig. 2a,b). These cells were generally low in abundance (1–5% of total cells taking up leucine) and were essentially absent in the samples where in situ pressure was maintained (Extended Data Table 1). Below, we operationally define the response of prokaryotic taxa as ‘piezosensitive’ if their activity is higher under depressurized conditions, ‘piezotolerant’ if the activity level under in situ pressure conditions is the same as under depressurized conditions and ‘piezophilic’ if the activity is higher under in situ than depressurized conditions. This highly active fraction detected under depressurized conditions can be considered the piezosensitive fraction of the prokaryotic community. Apparently, relieving these piezosensitive prokaryotes from hydrostatic pressure provoked the increase of bulk leucine incorporation rates. Analysing the changes of cell-specific uptake rates from in situ to atmospheric pressure conditions over the whole activity range allows estimation of the abundance of piezosensitive, piezotolerant and piezophilic prokaryotes. In the bathypelagic waters, 1–30% of cells taking up leucine were classified as piezosensitive (Extended Data Table 1). The majority (≥80%) of the deep-sea prokaryotes, however, were piezotolerant (Extended Data Table 1, except one sample). Only a small fraction (~5%) appeared to be piezophilic, exhibiting higher cell-specific activity under in situ pressure than under depressurized conditions (Extended Data Table 1). Only in one sample from 4,000 m depth had ~20% of the cells considered piezophilic (Extended Data Table 1). Leucine uptake rates of the piezophiles were generally low and never exceeded the uptake of the piezosensitive fraction.

**Extended Data Fig. 4:**
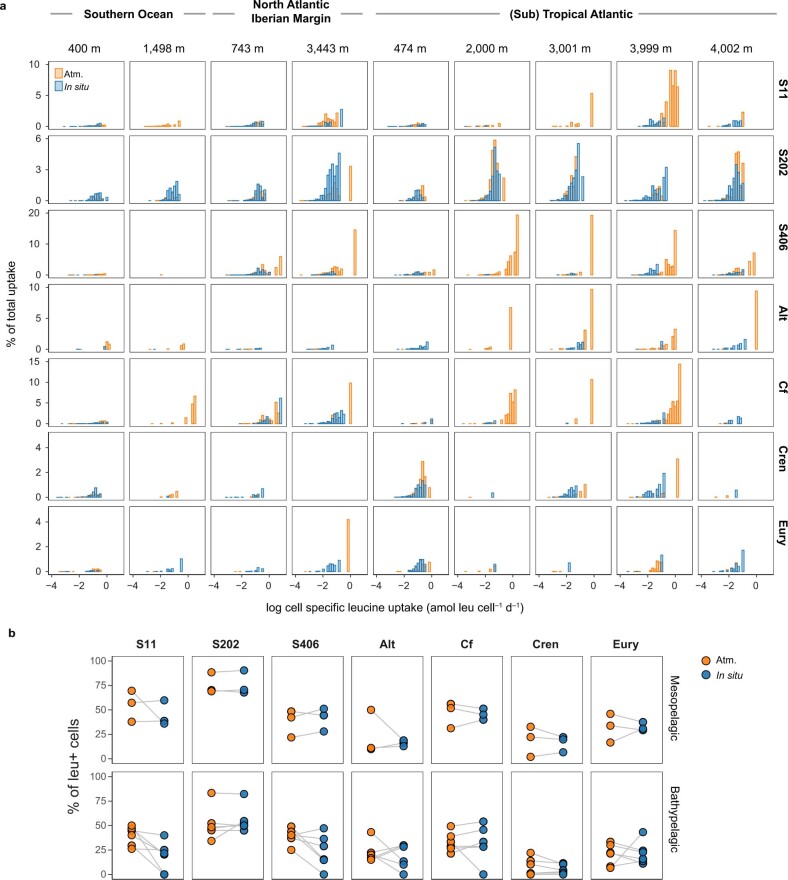
Taxon level response to the hydrostatic pressure. **a**, Cell specific leucine uptake incubated under in situ and atmospheric pressure (Atm.) conditions expressed as percentage of total leucine uptake. **b**, Abundance of cells taking up leucine in percent of total abundance of the respective taxon. Target group are indicated as S11: SAR11, S202: SAR202 clade, S406: SAR406 clade, Alt: *Alteromonas*, Cf: Bacteroidetes, Cren: Thaumarchaeota, Eury: Euryarchaeota. Source data

**Extended Data Fig. 5:**
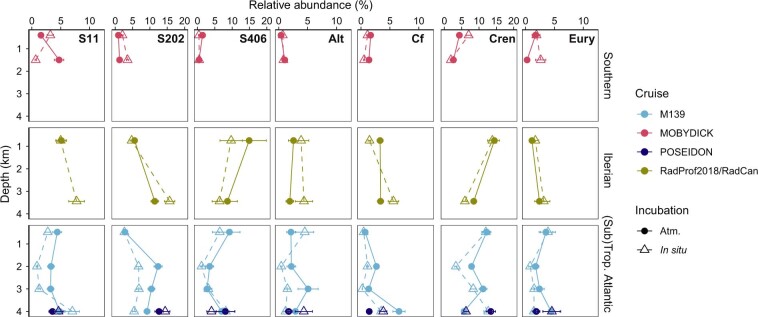
Relative abundance of prokaryotes. Error bar shows variations of technical and biological replicates calculated with coefficient of variations (CV). Randomly chosen technical and biological replicates for each taxonomic group were used to calculate the CV of relative abundance for the target groups; S11: SAR11 clade (n = 9), S202: SAR202 clade (n = 7), S406: SAR406 clade (n = 7), Alt: *Alteromonas* (n = 8), Cf: Bacteroidetes (n = 3), Cren: Thaumarchaeota (n = 11), Eury: Euryarchaeota (n = 8). Mean value of the CV was used to estimate the error.

Significantly higher heterotrophic activity upon depressurization, hence a piezosensitive response, was observed for several members of the bacterial community, particularly in Bacteroidetes (paired *t*-test, *n* = 18, one-sided *P* = 0.013), SAR406 (Marinomicrobia; Wilcoxon signed-rank test, *n* = 18, one-sided *P* = 0.002) and *Alteromonas*, especially from bathypelagic waters (paired *t*-test, *n* = 12, one-sided *P* = 0.006; Fig. 2c and Extended Data Fig. 4a). In contrast to these piezosensitive prokaryotes, SAR202 (Chloroflexi) showed no significant differences in leucine uptake between in situ and atmospheric pressure conditions (Wilcoxon signed-rank test, *n* = 18, *P* = 0.734 and *P* = 0.496 for SAR202 leucine uptake rates and relative abundance of SAR202 taking up leucine, respectively; Fig. 2c and Extended Data Fig. 4), indicative of a piezotolerant lifestyle. Thaumarchaeota contributed ~10% to the total prokaryotic abundance (Extended Data Fig. 5). However, only small fraction of Thaumarchaeota in the bathypelagic waters (~10% of thaumarchaeal cells) took up leucine under both in situ and atmospheric pressure conditions (Extended Data Fig. 4b). No difference in leucine uptake rates in Thaumarchaeota under in situ and atmospheric pressure was observed (Wilcoxon signed-rank test for leucine uptake, *n* = 18, *P* = 0.834; paired *t*-test for relative abundance of cells taking up leucine, *n* = 18, *P* = 0.148; Fig. 2c and Extended Data Fig. 4), indicating that bathypelagic Thaumarchaeota are probably piezotolerant. It should be noted, however, that the oligonucleotide probes used are targeting specific prokaryotic groups, which consist in turn of a mixture of different phenotypes with potentially different responses to hydrostatic pressure.

Taken together, we conclude that the vast majority of the deep-sea prokaryotic community is probably piezotolerant and only minor fractions are piezosensitive and piezophilic. While members of the Bacteroidetes and *Alteromonas* as well as the genus *Colwellia* have been shown to be piezophilic^20,21^, we consistently found that both Bacteroidetes and *Alteromonas* are piezosensitive. This might indicate that members of both taxa originate from surface waters and are associated with particles sinking out of the surface layers into the ocean’s interior. To obtain a better insight into the metabolic response of deep-sea prokaryotes upon depressurization, the metaproteome of abundant piezosensitive and piezotolerant bacterial taxa was analysed.

## Depth-related changes in the metaproteome

**Fig. 3:**
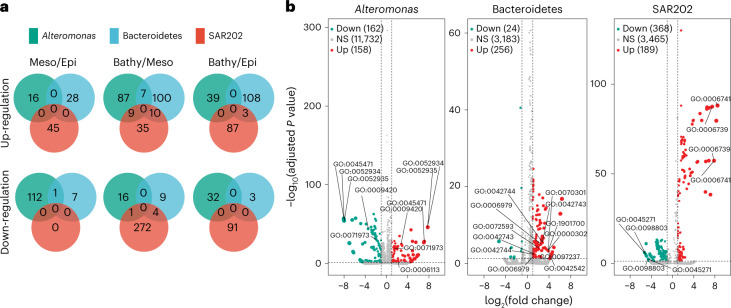
Depth-related changes in the metaproteome of three abundant deep-sea bacterial taxa. **a**, Venn diagrams indicating the number of shared and unique up- and down-regulated proteins among *Alteromonas*, Bacteroidetes and SAR202 of meso- versus epipelagic layers, bathy- versus mesopelagic layers and bathy- versus epipelagic layers. Numbers indicate the protein abundance. Epi, epipelagic; Meso, mesopelagic; Bathy, bathypelagic waters. **b**, Comparison of expressed proteins produced by *Alteromonas*, Bacteroidetes and SAR202. Significance of the change between depth layers is indicated by different colours: not significant (NS), *P* ≥ 0.05; up-regulated proteins (Up), *P* < 0.05 and log_2_ fold change ≥1; down-regulated proteins (Down), *P* < 0.05 and log_2_ fold change ≤ −1. The *P* values are shown in Supplementary Data 1.

While protein expression and function are influenced by hydrostatic pressure, monomeric proteins are rather stable compounds under a moderate pressure range (<400 MPa)^22^. Protein synthesis requires a certain period of time via transcription and translation, and is related to the growth rate of heterotrophic prokaryotes in the ocean^23^. Owing to the generally low growth rates of the heterotrophic prokaryotic community in the deep sea^13^, proteins extracted from deep-sea prokaryotes were probably expressed under in situ conditions. We performed metaproteomic analyses with a focus on *Alteromonas*, Bacteroidetes and SAR202 because single-cell activity measurements indicated that the former two bacterial taxa are piezosensitive while SAR202 is piezotolerant (Fig. 2c and Extended Data Fig. 4). We aimed at deciphering strategies of these different taxa to adapt to hydrostatic pressure. Based on gene ontology^24^, there is apparently no universal adaptation among these three bacterial taxa related to changes in hydrostatic pressure (Fig. 3a). However, taxa-specific differences were detectable related to the sampling depth and, hence, hydrostatic pressure (Fig. 3b and Supplementary Data 1).

Bacteroidetes up-regulated the response to oxidative stress (that is, response to hydrogen peroxide, reactive oxygen species and oxygen-containing compounds; Supplementary Data 1) in the bathypelagic as compared with the epipelagic layer. Culture-based analyses revealed that resistance against oxidative stress in deep-sea prokaryotes is an adaptation to high hydrostatic pressure and low temperature^25^. Particle-associated Bacteroidetes were suggested to exhibit a piezosensitive lifestyle^26^. Also, *Alteromonas* living in the bathypelagic realm exhibit a pronounced tendency towards a particle-associated lifestyle^27^. This particle-associated lifestyle of *Altermonas* in the bathypelagic layers allows them to access organic matter at higher concentrations on particles than in the ambient water^28^. Moreover, particles, such as deep-sea marine snow^29^, provide a micro-environment potentially favouring fermentation (that is, anaerobic respiration). *Alteromonas* showed flexibility to the change of hydrostatic pressure by down- and up-regulating the same genes depending on the depth layers (for example, GO:0045471, GO:0052934, GO:0052935, GO:0009420, GO:0071973; Fig. 3b, Supplementary Data 1). In the bathypelagic compared with the mesopelagic layers, the flagellum synthesis (GO:0009420, GO:0071973; Supplementary Data 1) and the fermentation pathway were up-regulated in *Alteromonas*. The response to ethanol (GO:0045471; Supplementary Data 1) was also up-regulated in the bathypelagic realm, probably related to the fermentation of algal-derived polysaccharides. In addition, the up-regulation of alcohol dehydrogenase activity (GO:0052934, GO:0052935) suggests counteracting oxidative damage due to high pressure and low temperature in bathypelagic *Alteromonas*.

In SAR202, NADP biosynthesis and metabolism (GO:0006741, GO:0006739) were up-regulated in the bathypelagic realm, while the respiratory chain complex 1 (GO:0045271, GO:0098803) was fivefold down-regulated compared with the mesopelagic realm (Supplementary Data 1). Respiratory chains are known to be affected by hydrostatic pressure, including in piezophilic bacteria^30^. This might be interpreted as an adaptation to the limited substrate availability in bathypelagic compared with the mesopelagic waters. It might be a strategy allowing for a similar activity under in situ and depressurized conditions as revealed by single-cell analysis (Extended Data Fig. 4a).

Taken together, there are taxa-specific modifications in *Alteromonas* and Bacteroidetes at the proteome level in bathypelagic cells, probably resulting in a higher energy expenditure to maintain a certain level of metabolism under high hydrostatic pressure as described in a previous study^31^. Upon depressurization, these specific adaptations are not required, leading overall to a higher metabolic activity of *Alteromonas* and Bacteroidetes under atmospheric pressure than at deep-sea pressure conditions. In contrast, SAR202 as a representative of the vast majority of piezotolerant prokaryotes down-regulates the respiratory complex 1 and up-regulates NADP biosynthesis in the bathypelagic realm to maintain the metabolic activity level under contrasting hydrostatic pressure conditions.

## Vertical transport of prokaryotes through the water column

Our results from single-cell analyses and metaproteomics support the conclusion that the piezosensitive microbes (mainly *Alteromonas* and Bacteroidetes) most likely originated from the upper water column. These piezosensitive bacteria instantly responded to the depressurization within the relatively short incubation period required to measure heterotrophic activity (3–12 h). Occasionally, the fraction of piezosensitive cells of the total active community was high (20–30%; Extended Data Table 1), tentatively indicating episodically rapid transport of these cells on sinking particles such as marine snow. *Alteromonas* and Bacteroidetes are known to be ubiquitous, generalistic/opportunistic bacterial taxa; the former are capable of rapidly exploiting available substrate^28^ and are abundant in marine snow from euphotic to bathypelagic waters^32^. Bacteroidetes are abundant in particle-rich epipelagic waters utilizing preferentially high-molecular-weight organic matter associated with phytoplankton blooms^28,33–35^ and are found on sinking particles at bathypelagic depth during elevated particle export events^36^. Hence, these bacterial taxa are probably transported from the surface to the deep waters via sedimenting particles.

The stimulation of heterotrophic prokaryotic activity under atmospheric pressure conditions could be caused by the release of intracellular organic matter from organisms upon depressurization. If we assume a prokaryotic carbon content^37,38^ of 10 fg C cell^−^^1^, the mean prokaryotic cell abundance in the bathypelagic waters (2.9 ± 1.4 × 10^4^ cells ml^−^^1^, *n* = 4; Extended Data Table 1) would result in 0.29 ± 0.14 μg C biomass l^−^^1^. The difference of the bulk heterotrophic bacterial biomass production between in situ incubations and under atmospheric pressure conditions was 0.003–0.029 pmol Leu l^−^^1^ h^−^^1^. Using a conversion factor of 1.55 kg C biomass mol^−^^1^ leucine incorporated, which is at the high end of conversion factors for deep-sea heterotrophic prokaryotes^39^, and a growth yield of 50%, this translates into an additional organic carbon demand of 0.43 ± 0.40 ng C l^−^^1^ (mean ± s.d., *n* = 4) under atmospheric as compared with in situ conditions. This is equivalent to 0.1–0.4% of the bathypelagic prokaryotic biomass. Thus, if only a few prokaryotic cells burst during the depressurization, it would be sufficient to stimulate heterotrophic prokaryotic activity under atmospheric pressure. No signs of cell debris, however, were noticed in microscopic examinations.

Other parameters potentially being altered upon depressurization are oxygen and carbon dioxide concentrations. Oxygen availability at all our study sites was not a growth limiting factor for aerobic prokaryotes (Supplementary Table 1) nor the changes in carbon dioxide concentrations and the associated small pH changes upon depressurization.

Regardless of whether or not some deep-sea prokaryotes released organic matter into the water or some physico-chemical parameters changed upon depressurization and thus provoked the higher metabolic activity of the bulk deep-sea heterotrophic prokaryotic community under atmospheric pressure, the major conclusion of our study remains: measuring deep-sea prokaryotic activity under atmospheric pressure conditions leads to a substantial overestimation of the actual in situ bulk prokaryotic activity. Consequently, deep-sea prokaryotic activity should be determined by maintaining the in situ hydrostatic pressure conditions to better constrain the deep-sea carbon flux^20,40,41^, as heterotrophic prokaryotes are by far the most important remineralizers of organic carbon in the ocean.

## Implication for the deep-sea carbon budget

**Extended Data Fig. 6:**
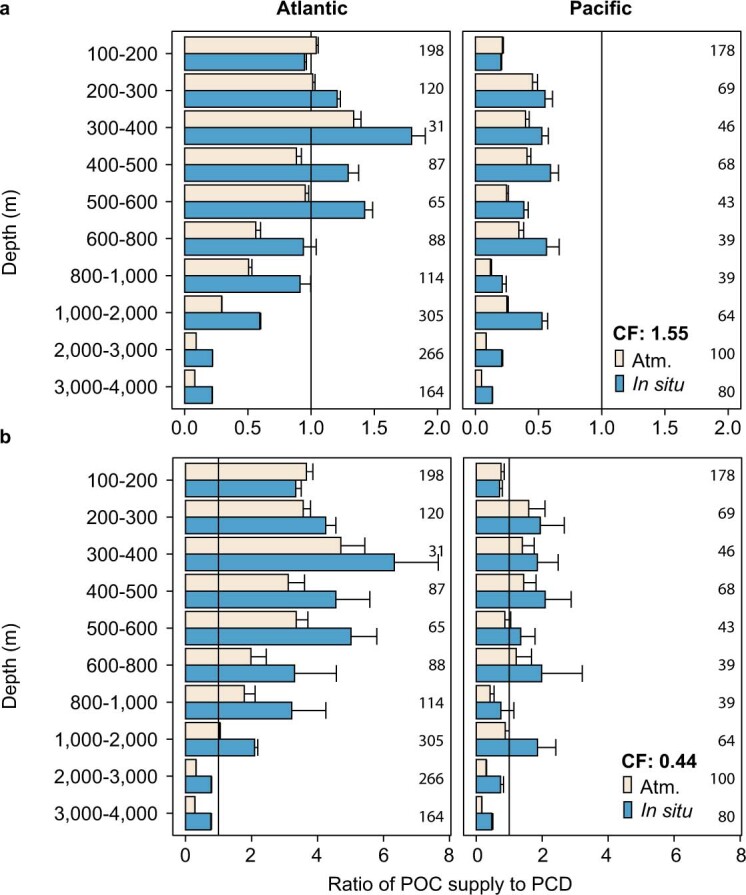
Ratio of modelled particulate organic carbon (POC) supply rate and prokaryotic carbon demand (PCD) calculated from depressurized and in situ heterotrophic production rates in the Atlantic and the Pacific Ocean. The particulate organic carbon (POC) potentially available at a specific depth is calculated using depth dependent sediment trap data^66^ and satellite derived net primary production estimates. The prokaryotic carbon demand assumes a grand average of 8% growth efficiency for the meso- and 3% for the bathypelagic waters. PCD was calculated using leucine to carbon conversion factors (CF) of **a**, 1.55 kg C mol^–1^ leu and **b**, 0.44 kg C mol^–1^ leu (see Methods). A ratio of 1 indicates that the POC supply rate matches PCD. Values <1 suggest inadequate supply of POC to support the PCD. Error bars indicate standards errors of the mean taking error propagation into account. Numbers in the panels indicate sample size.

Apparently, heterotrophic biomass production of deep-sea prokaryotes has been overestimated in the past, as almost all the estimates have been based on measurements performed under atmospheric pressure conditions^1,9^. It is likely that the biomass production and respiration of the bulk prokaryotic community are reduced proportionally under in situ pressure conditions. Hence, the growth efficiency remains probably unaffected under in situ pressure conditions. The heterotrophic PCD (sum of carbon biomass production and respiration) at several depth horizons of the ocean water column can be compared with the estimated particle flux into the ocean’s interior using heterotrophic prokaryotic production (see Methods). Assuming a growth efficiency of 8% and 3% for meso- and bathypelagic layers, respectively^13,42^, and applying the leucine-to-carbon conversion factors of 1.55 and 0.44 kg C mol^−^^1^ leucine incorporated^42,43^, the estimated PCD obtained from in situ activity measurements and the POC supply is largely balanced (Extended Data Fig. 6). Moreover, there are neutrally buoyant or slowly sinking detrital particles laterally transported through the water column and not, or only very inefficiently, collected by sediment traps presenting an additional source of organic carbon for heterotrophic microbes^9^, as well as organic matter production by chemolithoautotrophs^38^. The extent to which these two sources of organic carbon contribute to the carbon requirements of heterotrophic prokaryotes in the deep sea remains unknown^9^. A conversion factor of 0.44 kg C mol^−^^1^ leucine incorporated has been reported for heterotrophic mesopelagic prokaryotic communities^39,44^. Hence, it is likely that this conservative conversion factor is closely reflecting the actual PCD in the bathypelagic realm; however, uncertainties in the validity of this conversion factor remain.

Our study shows that the bulk prokaryotic heterotrophic activity in the deep sea is substantially inhibited by the hydrostatic pressure in the meso- and bathypelagic realm of the ocean. Thus, despite the fact that the prokaryotic community composition is depth-stratified^45^, the small fraction (~10%) of piezosensitive prokaryotes transported into the deep ocean via particle sedimentation can strongly affect bathypelagic heterotrophic prokaryotic activity measurements performed under atmospheric pressure conditions. Also, only a rather minor fraction (about 5%) appears to be piezophilic in the bathypelagic ocean.

Overall, by taking the inhibitory effect of hydrostatic pressure on the metabolism of the bulk deep-sea heterotrophic prokaryotic community into consideration, the heterotrophic PCD and POC supply appears to be largely balanced in the global ocean’s interior. Hence, the reported mismatch between organic carbon supply and prokaryotic carbon demand in the bathypelagic realm is probably largely due to an overestimation of the heterotrophic prokaryotic activity when measured under atmospheric pressure conditions. Our findings of reduced prokaryotic heterotrophic activity under the high-pressure conditions in the deep sea might have important implications for geo-engineering strategies such as delivery of organic carbon to the deep sea to mitigate the carbon dioxide increase in the atmosphere.

## Methods

### Collecting and incubating samples at in situ hydrostatic pressure and under atmospheric conditions

For measuring heterotrophic biomass production under in situ hydrostatic pressure conditions, water samples were collected and incubated with the autonomous ISMI (NiGK corporation; Extended Data Fig. 2). The ISMI is lowered via a winch from the research vessel to the pre-defined depth. The ISMI is a programmable device consisting of 500 ml polycarbonate incubation and fixation bottles and peristaltic pumps (~150 ml min^−^^1^). These parts are connected by silicone tubing. All parts in direct contact with the water samples were thoroughly cleaned (see below). After lowering the ISMI to the pre-defined depth, ambient seawater was pumped by the peristaltic pump into duplicate or triplicate incubation bottles to which ^3^H-leucine (5 nM final concentration, 10 nM for epipelagic samples of the Southern Ocean, [3,4,5-^3^H] L-leucine with a specific activity ranging between 110 and 120 Ci mmol^−^^1^, either from Biotrend or PerkinElmer) was added prior to deployment. The saturating substrate concentrations were determined for each biogeographic province on samples collected at the respective depth. Immediately after filling the polycarbonate bottles, subsamples (~100 ml) were transferred from the incubation bottles to the fixation bottles containing 0.2 µm filtered formaldehyde (final concentration 2%) to serve as a killed control (T0), while the live samples were fixed with 2% formaldehyde (final conc.) after 3–12 h of incubation at the respective incubation depth (Supplementary Table 2) at the end of the incubation (Tf) according to the pre-programmed incubation time. All the incubation bottles and tubes in contact with the sample were stored in 0.4–0.5 N HCl overnight, washed three times with Milli-Q water and rinsed three times with the corresponding 0.2 µm filtered seawater prior to the deployment. The performance of the ISMI has been extensively tested. No significant difference in leucine incorporation was observed between the complete setup of the ISMI and detached ISMI bottles (as a control under atmospheric pressure condition). ^3^H-leucine in the bottles was homogenously distributed as determined in previous tests.

For comparing heterotrophic prokaryotic production under in situ pressure with that under atmospheric pressure, water samples were collected at the same depth and within 2–4 h of the deployment of the ISMI using Niskin bottles mounted on a conductivity–temperature–depth (CTD) rosette system (Supplementary Table 1). The hoisting speed of the CTD was 1.0 m s^−^^1^. Water samples were collected immediately after the CTD arrived on deck of the research vessel and kept in an incubator or water-bath at the respective in situ temperature of the sampling depth. The temperature of the water samples collected from the Niskin bottles was typically 2–3 °C higher than the in situ temperature. Thus, the incubation bottles were incubated for 1–3 h prior to the incubation to attain the in situ temperature again (Supplementary Table 2). Sampling of the nepheloid layer was avoided as indicated by the signals of the transmissometer and the optical backscattering sensors mounted on the CTD.

Incubations at atmospheric pressure were performed in identical polycarbonate bottles as used for in situ incubations. Three live subsamples and two formaldehyde killed (2% final conc.) controls were used per sample (see Supplementary Table 2) and incubated in temperature-controlled chambers at the same temperature as the in situ samples (Supplementary Table 2). Although samples were incubated under atmospheric pressure conditions in the same incubation bottles as used in the ISMI, samples have been collected at different times potentially resulting in collecting water with subtle differences in the chemical and microbiological characteristics. Although we cannot rule out that this might have biased our results, it is unlikely that this had a major influence on the results and the conclusion of this study.

### Bulk heterotrophic prokaryotic biomass production measurements

Leucine incorporation rates were determined according to ref. ^17^. Following formaldehyde fixation of the live samples, samples and controls from in situ and atmospheric pressure incubations were filtered onto 0.2 µm polycarbonate filters (25 mm filter diameter, Nuclepore, Whatman). Subsequently, the filters were rinsed twice with 5% ice-cold trichloroacetic acid and twice with Milli-Q water. Filters were air-dried and placed in scintillation vials. Then, 8 ml of scintillation cocktail (either Filter-Count or Ultima Gold, PerkinElmer, depending on the research expedition) was added. After about 16 h, the samples were counted in a liquid scintillation counter (Packard, Tri-Carb) onboard, and the disintegrations per minute obtained were converted into bulk leucine incorporation rates. Additionally, the disintegrations per minute in 10 µl sample water were determined to check the final concentration of leucine in the incubation vessels of the ISMI.

### MICRO–CARD–FISH

For microautoradiography combined with catalysed reporter deposition fluorescence in situ hybridization (MICRO–CARD–FISH), live samples and formaldehyde-fixed (2% final conc.) controls were incubated at in situ and atmospheric pressure conditions as described above. After an incubation time of 3–12 h (Supplementary Table 2), the live samples were fixed with formaldehyde. Upon hoisting the ISMI onboard the research vessel, the water contained in the polycarbonate bottles and the samples from the incubations under atmospheric pressure conditions were filtered onto 0.2 µm polycarbonate filters (25 mm filter diameter, GTTP, Millipore) and rinsed twice with Milli-Q water. After drying, the filters were stored at −20 °C until further processing. At the home laboratory, the filters were processed^46^. To permeabilize archaea, filters were incubated in 0.1 M HCl^47^. Samples were hybridized (at 35 °C for 15 h and washing at 37 °C for 15 min) with horseradish peroxidase labelled oligonucleotide probes (Supplementary Table 3) and amplified with Alexa Fluor 488 tyramide at 46 °C for 15 min. After CARD–FISH, the filters were embedded in photographic emulsion (K5, ILFORD) and exposed at 4 °C for 14 days in the dark with silica gel as a drying agent. Development and fixing were performed according to the manufacturer’s instructions (developer: Phenisol, ILFORD; fixer: Hypam, ILFORD). Samples were counterstained with 4′,6-diamidino-2-phenylindole (DAPI). Slides were examined on an epifluorescence microscope (Axio Imager M2, Carl Zeiss) equipped with the appropriate filter sets and a camera for photo capturing (≥10 fields). More than 1,000 DAPI-stained cells were enumerated for each CARD–FISH sample. All samples were also hybridized with the antisense probe NON388 (Supplementary Table 3) for unspecific hybridization control. Unspecific binding was always <1% of DAPI-stained cells. Total active cells analysed per sample amounted to: for the mesopelagic cells, *n* ≥ 6,478 at in situ and *n* ≥ 6,555 under atmospheric pressure conditions; for bathypelagic cells, *n* ≥ 2,162 at in situ and *n* ≥ 1,788 under atmospheric pressure conditions. Cell-specific activity of the different target prokaryotic groups was analysed by sizing the silver grain halo surrounding probe-positive cells using Axio Vision SE64 Re4.9 (Carl Zeiss). The size of the silver grain area around a cell was converted to single-cell leucine uptake rate (amol Leu cell^−^^1^ d^−^^1^) based on the regression^19^ obtained using our data set: *R*_halo_ = 9.72 × 10^7^ *R*_leu_ (*r*^2^ = 0.96), where *R*_leu_ is leucine incorporation rate (pmol Leu l^−^^1^ h^−^^1^) and *R*_halo_ is the total silver grain halo volume (µm^3^ l^−^^1^ h^−^^1^) calculated from the area size of the silver grain assuming a spherical distribution. The relatively weak radiation of tritium creates a hemisphere distribution around the cells taking up ^3^H-leucine in the emulsion. Consequently, we calculated the volume of the halo rather than the area (Supplementary Fig. 1). The distribution of cell-specific activities was first expressed as a histogram with a bin interval of 0.17 (amol Leu cell^−^^1^ d^−^^1^ in log_10_ scale calculated with the smallest number of counts: *n* = 1,788) determined by the kernel estimation based approach^48^. Subsequently, the histogram was used to determine the abundances of piezosensitive, piezotolerant and piezophilic prokaryotes. Cells with specific activities assigned to the same bin were considered to have the same activity. Therefore, when cell-specific uptake rates were classified in the same bin in both in situ and atmospheric pressure conditions, these cells were assigned as piezotolerant. Piezosensitive cells were determined as those cells altering their activity from lower to higher activity bins upon depressurization, and their minimum and maximum abundances were determined. Accordingly, piezophilic cells were those shifting in the activity bins from higher to lower activity upon depressurization.

### Construction of metagenomic assembled genomes

We used metagenomic assembled genomes (MAGs) to construct a comprehensive gene catalogue for the selected taxa with metagenomic reads using the data set of the *Tara Ocean* and *Malaspina* cruise as well as MAGs from previous publications^49–51^. The paired-end reads from each metagenome were assembled using MEGAHIT v.1.1.1 (*k* list: 21, 29, 39, 59, 79, 99, 119, 141)^52^. The contigs were clustered with two separate automatic binning algorithms: MaxBin^53^ and MetaBAT2^54^ with default settings. The generated genomic bins were de-replicated and refined with MetaWRAP (bin_refinement). Bins with >70% completeness and <10% contamination (−*c* 70, −*x* 10) were kept and pooled with publicly available MAGs^51^ for de-replication using dRep^55^. The phylogenetic affiliation of each MAG was determined using GTDB-Tk^56^. Bacteroidetes-like, *Alteromonas*-like and SAR202-like MAGs were selected as representatives for downstream analysis. Gene prediction was performed using Prodigal^57^. The predicted genes of each taxa were clustered using 90% similarity applying Cd-hit^58^ to construct a non-redundant protein database, which was used for metaproteomic analysis.

### Metaproteomic analyses of selected bacterial taxa

Metaproteomic data were retrieved from a previous study^28^. Samples for metaproteomic analyses were collected either by Niskin bottles or by in situ pumps (WTS-LV, McLane) with 0.2 µm polycarbonate filters mounted^28^. Metaproteomics data were pooled into three groups (epi-, meso- and bathypelagic) according to depth. The tandem mass spectrometry spectra from each proteomic sample were searched against the taxa-specific non-redundant protein database using SEQUEST engines^59^ and validated with Percolator in Proteome Discoverer 2.1 (Thermo Fisher Scientific). To reduce the probability of false peptide identification, the target-decoy approach^60^ was used and results <1% false discovery rate at the peptide level were kept. Qualified results from peptide–spectrum matches were used for metaproteomic gene ontology enrichment analysis^61^ (MetaGOmics, https://www.yeastrc.org/metagomics/home.do) according to the instructions. Gene ontology terms with |log_2_ fold change| ≥1 and adjusted *P* value of <0.05 were identified as differentially expressed when comparing samples from different depth layers.

### Calculating the potentially available POC and PCD

For estimating the ratio between PCD and POC supply, we assembled a large database of prokaryotic ^3^H-leucine incorporation measurements in the Atlantic (*n* = 1,440) and the Pacific (*n* = 783)^13,62–65^. Prokaryotic heterotrophic production (PHP) was calculated using the leucine-to-carbon conversion factor of 1.55 kg C mol^−^^1^ leucine^43^ and 0.44 kg C mol^−^^1^ leucine^42^. There are higher and lower conversion factors published; however, for our basin-wide production data, the applied conversion factors represent the extremes found for specific sites^39^. To calculate PHP rates more typical for in situ pressure conditions, we applied the power law fit of Fig. 1 to the measurements performed under atmospheric pressure conditions: PHP_in situ_ = (PHP_atm_ × 494 × *z*^−^^0.321^)/100, where *z* is depth (metres) and PHP_in situ_ and PHP_atm_ are in µmol C m^−^^3^ d^−^^1^ under in situ and atmospheric pressure conditions, respectively. With these data, the PCD was calculated as PCD = PHP/PGE. From publicly available data, a median prokaryotic growth efficiency (PGE) of 8% was applied^42^. A similar value was also reported for the mesopelagic waters in the North Pacific^66^. Consequently, we used a PGE of 8% for mesopelagic depths and a PGE of 3% for bathypelagic waters^13^.

The POC potentially available at a specific depth (POC_a_) was calculated by POC_a_ (mmol m^−^^3^ d^−^^1^) = 0.2 × NPP^1.66^ × *z*^−^^1.68^. The algorithm is based on thorium-corrected sediment trap data from the North Atlantic spanning all major biomes^67^, where NPP is the net primary production and *z* is the depth in the water column for which the POC input per day is calculated. The original model calculates fluxes in g C m^−^^2^ yr^−^^1^, which we converted to mmol C m^−^^3^ d^−^^1^ to allow comparison of daily rates of PCD with POC input into the specific depth layers. NPP was obtained from the Ocean Productivity website (http://www.science.oregonstate.edu/ocean.productivity) and derived from the Vertically Generalized Production Model (VGPM)^68^ using satellite eight-day averages of chlorophyll. NPP data on the 0.2 × 0.2° grid were matched to the nearest degree in longitude and latitude of the stations and the time of sampling for heterotrophic prokaryotic production.

### Analysis and presentation

Statistics and graphics in this study were performed with R version 4.1.1 using RStudio version 1.4.1717 and GMT version 5.4.1. For paired sample tests, normality was checked with the Shapiro–Wilk test. If data were normally distributed, a *t*-test was performed, otherwise non-parametric tests were applied. If not specified, a two-sided test was performed.

## Online content

Any methods, additional references, Nature Portfolio reporting summaries, source data, extended data, supplementary information, acknowledgements, peer review information; details of author contributions and competing interests; and statements of data and code availability are available at 10.1038/s41561-022-01081-3.

### Supplementary information

Supplementary Information

Supplementary Data 1 List of up- and down-regulated functions of *Alteromonas*, Bacteroidetes and SAR202 using gene ontology,

### Source data

Source Data Fig. 1 csv file containing the embedded data

Source Data Fig. 2a,c Excel file containing the embedded data

Source Data Fig. 2b Microscopic image

Source Data Extended Data Fig. 4 csv file containing the embedded data

## Figures and Tables

**Extended Data Table 1:**
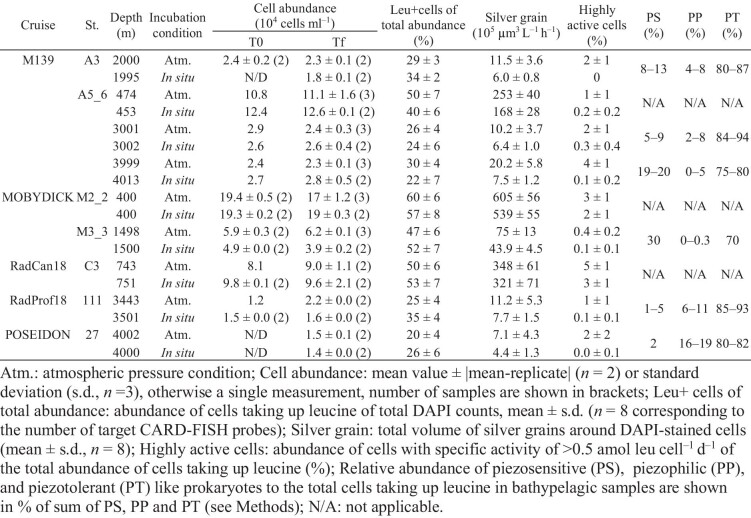
Abundance of cells taking up ^3^H-leucine under in situ and atmospheric pressure conditions as determined by MICRO-CARD-FISH

## Data Availability

Data supporting the findings of this study are available in the paper and its Supplementary Information files. Station information of the following research cruises is available at the following websites: for the research cruise SO248 (10.1594/PANGAEA.864673); for M139 (10.1594/PANGAEA.881298); and for MOBYDICK (http://www.obs-vlfr.fr/proof/php/mobydick/mobydick.php). Source data are provided with this paper.
