# Supplementary material for: Limited carbon cycling due to high-pressure effects on the deep-sea microbiome
Source: Nat Geosci. 2022 Nov 28;15(12):1041–7. doi: 10.1038/s41561-022-01081-3 (PMC9726642; doi:10.1038/s41561-022-01081-3)
Supplement: Supplementary file 1 — Supplementary Information [file 41561_2022_1081_MOESM1_ESM.pdf]

nature geoscience

Article https://doi.org/10.1038/s41561-022-01081-3

Limited carbon cycling due to high-pressure effects on the deep-sea microbiome

In the format provided by the authors and unedited

Supplementary information

Supplementary Table 1. Station details on prokaryotic activity measurements performed in this study. Information is provided for the on-deck atmospheric pressure samples collected by the sampling rosette with the conductivity-temperature-depth (CTD) system.

Cruise or sampling site	Area	St	Date	Latitude (°)		Longitude (°)		Depth (m)	Temp (°C)	Salinity	DO (μmol kg^{-1})
MODUPLAN	North Atlantic Ocean	8	5-Aug-2014	43.0000	N	9.7165	W	445	11.39	35.67	189
MODUPLAN	North Atlantic Ocean	14	8-Aug-2014	42.9998	N	10.6667	W	1140	10.60	36.12	171
MODUPLAN	North Atlantic Ocean	108	10-Aug-2014	43.0000	N	12.6667	W	1778	4.35	35.05	233
MODUPLAN	North Atlantic Ocean	59	19-Aug-2014	43.9000	N	3.7833	W	1974	3.91	35.03	229
MODUPLAN	North Atlantic Ocean	60	20-Aug-2014	43.8000	N	3.7833	W	990	9.72	35.78	175
MODUPLAN	North Atlantic Ocean	57	21-Aug-2014	44.2917	N	3.7832	W	1975	4.10	35.04	228
Rovinj*	Adriatic Sea	N/A	25-Oct-2014	45.0856	N	13.6394	E	20	19.50	N/D	N/D
RadProf15	North Atlantic Ocean	11	1-Aug-2015	43.0253	N	10.0173	W	2465	3.14	34.96	220
RadProf15	North Atlantic Ocean	111	5-Aug-2015	43.0010	N	14.0328	W	3152	2.79	34.94	213
SO248	Pacific Ocean	16†	26-May-2016	49.9999	N	179.5501	E	999	2.73	34.36	26
SO248*	Pacific Ocean	17	29-May-2016	54.0013	N	179.5813	E	501	3.63	34.08	26
M139*	Atlantic Ocean	A1†	12-Jul-2017	15.8860	N	68.9148	W	2002	4.12	34.98	197
M139	Atlantic Ocean	A3†	22-Jul-2017	23.5538	N	48.0838	W	3998	2.29	34.89	227
M139	Atlantic Ocean	A3†	22-Jul-2017	23.5538	N	48.0838	W	2000	3.68	35.00	223
M139	Atlantic Ocean	A5_6†	31-Jul-2017	10.3388	N	36.9597	W	3999	2.33	34.89	221
M139	Atlantic Ocean	A5_6†	31-Jul-2017	10.3388	N	36.9597	W	3001	2.75	34.93	225
M139	Atlantic Ocean	A5_6†	31-Jul-2017	10.3392	N	36.9617	W	474	8.56	34.90	69
MOBYDICK	Southern Ocean	M2_1†	26-Feb-2018	50.6160	S	72.0010	E	448	2.25	34.38	194
MOBYDICK	Southern Ocean	M3_1†	3-Mar-2018	50.6835	S	68.0622	E	1499	2.00	34.76	190
MOBYDICK	Southern Ocean	M2_2†	6-Mar-2018	50.6252	S	72.0134	E	400	2.16	34.30	211
MOBYDICK*	Southern Ocean	M1	8-Mar-2018	49.8502	S	74.9017	E	2499	1.01	34.72	197
MOBYDICK	Southern Ocean	M2_3	16-Mar-2018	50.6159	S	72.0013	E	175	1.72	33.99	302
MOBYDICK	Southern Ocean	M3_3	18-Mar-2018	50.6877	S	68.0664	E	1498	2.07	34.76	188
RadProf18	North Atlantic Ocean	111	24-Aug-2018	42.9998	N	14.0350	W	3443	2.63	34.93	224
RadCan18	North Atlantic Ocean	C3	27-Aug-2018	43.7668	N	6.1672	W	743	10.23	35.67	182
POSEIDON	Atlantic Ocean	5	7-Mar-2019	36.1575	S	44.3407	W	2565	2.41	34.82	204
POSEIDON	Atlantic Ocean	9	12-Mar-2019	25.4064	S	35.0020	W	3156	2.66	34.91	237
POSEIDON	Atlantic Ocean	13	16-Mar-2019	14.4742	S	30.6714	W	2962	2.69	34.90	229
POSEIDON	Atlantic Ocean	19	23-Mar-2019	5.9091	N	29.7460	W	2375	3.16	34.95	229
POSEIDON	Atlantic Ocean	22	26-Mar-2019	13.5957	N	29.7139	W	3942	2.37	34.89	218
POSEIDON	Atlantic Ocean	27	31-Mar-2019	23.7935	N	23.9188	W	4002	2.40	34.90	219

*Sample used for instrumental test; †Metaproteomics stations[1]; N/A: not applicable; N/D: not determined; DO-dissolved oxygen measured with an oxygen sensor (Sea-Bird SBE 43 or Aanderaa 4831F).

Supplementary Table 2. Leucine incorporation rates and incubation conditions under *in situ* and atmospheric pressure conditions.

Cruise/ sampling site	St.	Atmospheric pressure condition						*In situ* condition					
		Depth (m)	Inc. temp. (°C)	Duration (h)	Leu incorp. Mean	n	SD or mean – repl.	Depth (m)	Inc. temp. (°C)	Duration (h)	Leu incorp. Mean	n	SD or mean – repl.
MODUPLAN	8	445	10	6.2	0.400	1	N/A	500	11.4	6.0	0.289	2	0.035
	14	1140	10	8.0	0.088	2	0.000	1150	10.6	8.0	0.046	2	0.003
	108	1778	4	10.4	0.028	1	N/A	1800	4.4	9.5	0.013	2	0.006
	59	1974	4	8.1	0.077	1	N/A	2000	3.9	8.1	0.009	2	0.004
	60	990	10	8.2	0.074	2	0.032	1000	9.7	8.0	0.053	2	0.002
	57	1975	4	8.1	0.033	2	0.006	2000	4.1	7.7	0.008	2	0.002
RadProf15	11	2465	0–3	12.2	0.047	3	0.017	2500	3.1	11.8	0.011	2	0.004
	111	3152	2.5	12.1	0.014	3	0.010	3200	2.8	12.0	0.007	2	0.000
SO248	16	999	4	8.1	0.088	3	0.003	1000	2.7	7.5	0.058	2	0.018
M139	A3	3998	2.5	12.0	0.011	3	0.000	3983	2.3	12.0	0.003	3	0.000
	A3	2000	2.5	10.2	0.016	3	0.000	1995	3.7	10.0	0.004	3	0.001
	A5_6	3999	2.5	11.0	0.033	3	0.002	4013	2.3	10.6	0.004	2	0.000
	A5_6	3001	2.5	10.7	0.013	3	0.001	3002	2.8	11.0	0.004	2	0.000
	A5_6	474	7	6.0	0.336	3	0.022	453	8.6	6.0	0.205	2	0.018
MOBYDICK	M2_1	448	2.2	7.5	1.228	3	0.023	450	2.2	2.8	1.089	2	0.030
	M3_1	1499	1.9	7.0	0.070	3	0.002	1470	2.0	5.8	0.047	2	0.002
	M2_2	400	1.4	7.3	0.651	3	0.023	400	2.2	7.2	0.484	2	0.048
	M2_3	175	1.4	4.1	1.400	3	0.050	175	1.7	4.2	1.118	2	0.038
	M3_3	1498	1.7	7.2	0.060	3	0.008	1500	2.1	7.6	0.044	2	0.002
RadProf18	111	3443	2.6	8.3	0.007	3	0.000	3501	2.6	8.0	0.003	2	0.000
RadCan18	C3	743	10.2	3.3	0.440	3	0.004	751	10.2	3.3	0.243	2	0.006
POSEIDON	5	2565	2.4	7.6	0.023	2	0.002	2560	2.4	7.0	0.013	2	0.001
	9	3156	2.7	6.9	0.004	2	0.001	3155	2.7	6.8	0.003	2	0.000
	13	2962	2.7	6.8	0.006	2	0.001	2961	2.7	6.6	0.002	2	0.000
	19	2375	3.2	7.5	0.012	2	0.000	2375	3.2	7.2	0.004	2	0.001
	22	3942	2.4	8.2	0.005	2	0.000	3941	2.4	8.3	0.002	2	0.000
	27	4002	2.4	8.6	0.005	2	0.000	4000	2.4	8.4	0.001	2	0.000

Inc. temp.: incubation temperature; Duration: incubation time; Leu incorp.: leucine incorporation rate in pmol leu L^{-1} h^{-1}; mean – repl.: |mean-replicate| of duplicate bottle incubations; standard deviation (SD) was used when triplicates were available; N/A: not applicable.

Supplementary Table 3. Oligonucleotide probes applied in MICRO-CARD-FISH analyses.

Target organisms	Probe	Sequence (5′ to 3′)	FA (%)
Bacteria	EUB338[2]	GCTGCCTCCCGTAGGAGT	55
	EUB338-II[3]	GCAGCCACCCGTAGGTGT	
	EUB338-III[3]	GCTGCCACCCGTAGGTGT	
SAR11 clade	SAR11-152R[4]	ATTAGCACAAGTTTCCYCGTGT	45
	SAR11-441R[4]	TACAGTCATTTTCTTCCCCGAC	
	SAR11-542R[4]	TCCGAACTACGCTAGGTC	
	SAR11-732R[4]	GTCAGTAATGATCCAGAAAGYTG	
SAR202 clade	SAR202-312[5]	TGTCTCAGTCCCCCTCTG	55
SAR406 clade	SAR406-97[6]	CACCCGTTCGCCAGTTTA	60
Thaumarchaeota Marine Group I	Cren554[7]	TTAGGCCCAATAATCMTCCT	20
	Cren537[8]	TGACCACTTGAGGTGCTG	
Alteromonas/*Colwellia*	Alt1413[9]	TTTGCATCCCACTCCCAT	55
Bacteroidetes	CF319a[10]	TGGTCCGTGTCTCAGTAC	55
Euryarchaeota Marine Group II	Eury806[8]	CACAGCGTTTACACCTAG	20
Negative control Bacteria	NON338[11]	ACTCCTACGGGAGGCAGC	55

FA: Formamide concentration of the hybridization buffer

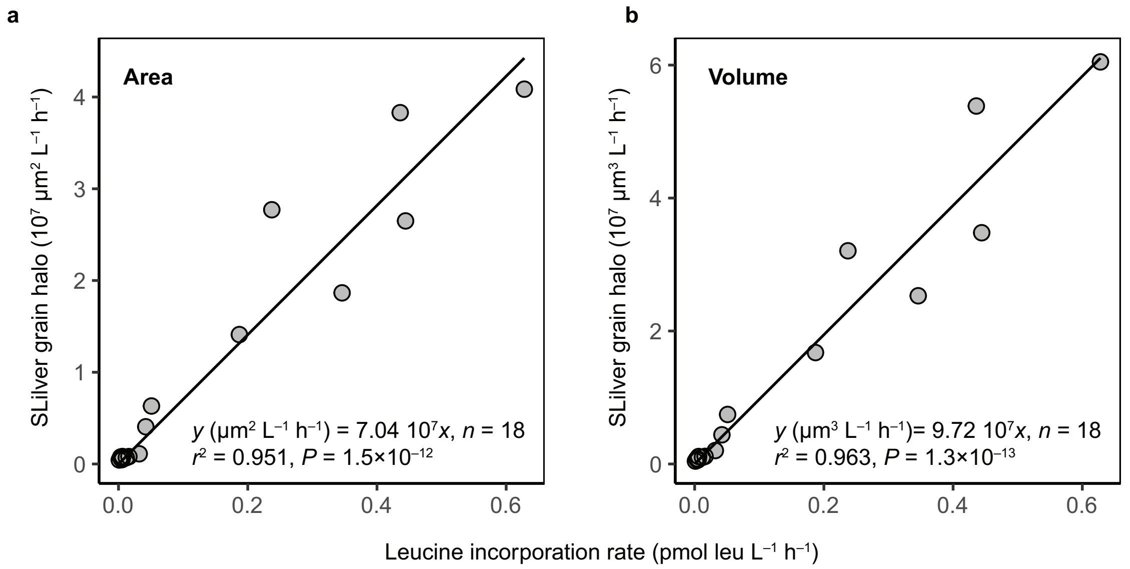

Supplementary Fig. 1. Single-cell activity measurements. Relationship of bulk leucine incorporation rates and silver grain halo around cells expressed as **a**, area and **b**, volume. All the MICRO-CARD-FISH samples under *in situ* and atmospheric pressure conditions at 9 stations (see Methods) were used to obtain the regressions. Plots are shown as mean values of 8 technical replicates which correspond to the number of probes used. A slightly better correlation between bulk leucine incorporation and silver grain halo volume than with halo area was found. Consequently, single-cell leucine uptake was calculated using the regression with halo volume in this study.

Supplementary references:

1 Zhao, Z., Baltar, F. & Herndl, G. J. Linking extracellular enzymes to phylogeny indicates a predominantly particle-associated lifestyle of deep-sea prokaryotes. *Sci. Adv.* 6, eaaz4354 (2020).

2 Amann, R. I. *et al.* Combination of 16S rRNA-targeted oligonucleotide probes with flow cytometry for analyzing mixed microbial populations. *Appl. Environ. Microbiol.* **56**, 1919–1925 (1990).

3 Daims, H., Bruhl, A., Amann, R., Schleifer, K. H. & Wagner, M. The domain-specific probe EUB338 is insufficient for the detection of all Bacteria: Development and evaluation of a more comprehensive probe set. *Syst. Appl. Microbiol.* **22**, 434–444 (1999).

4 Morris, R. M. *et al.* SAR11 clade dominates ocean surface bacterioplankton communities. *Nature* **420**, 806–810 (2002).

5 Morris, R. M., Rappe, M. S., Urbach, E., Connon, S. A. & Giovannoni, S. J. Prevalence of the *Chloroflexi*-related SAR202 bacterioplankton cluster throughout the mesopelagic zone and deep ocean. *Appl. Environ. Microbiol.* **70**, 2836–2842 (2004).

6 Fuchs, B. M., Woebken, D., Zubkov, M. V., Burkill, P. & Amann, R. Molecular identification of picoplankton populations in contrasting waters of the Arabian Sea. *Aquat. Microb. Ecol.* **39**, 145–157 (2005).

7 Massana, R., Murray, A. E., Preston, C. M. & DeLong, E. F. Vertical distribution and phylogenetic characterization of marine planktonic *Archaea* in the Santa Barbara Channel. *Appl. Environ. Microbiol.* **63**, 50–56 (1997).

8 Teira, E., Reinthaler, T., Pernthaler, A., Pernthaler, J. & Herndl, G. J. Combining catalyzed reporter deposition-fluorescence in situ hybridization and microautoradiography to detect substrate utilization by Bacteria and Archaea in the deep ocean. *Appl. Environ. Microbiol.* **70**, 4411–4414 (2004).

9 Eilers, H., Pernthaler, J., Glockner, F. O. & Amann, R. Culturability and in situ abundance of pelagic bacteria from the North Sea. *Appl. Environ. Microbiol.* **66**, 3044–3051 (2000).

10 Manz, W., Amann, R., Ludwig, W., Vancanneyt, M. & Schleifer, K. H. Application of a suite of 16S rRNA-specific oligonucleotide probes designed to investigate bacteria of the phylum cytophaga-flavobacter-bacteroides in the natural environment. *Microbiology* 142, 1097–1106 (1996).

11 Wallner, G., Amann, R. & Beisker, W. Optimizing fluorescent in situ hybridization with rRNA-targeted oligonucleotide probes for flow cytometric identification of microorganisms. *Cytometry* 14, 136–143 (1993).
