# Supplementary figures and images for: Limited carbon cycling due to high-pressure effects on the deep-sea microbiome
Source: Nat Geosci. 2022 Nov 28;15(12):1041–7. doi: 10.1038/s41561-022-01081-3 (PMC9726642; doi:10.1038/s41561-022-01081-3)

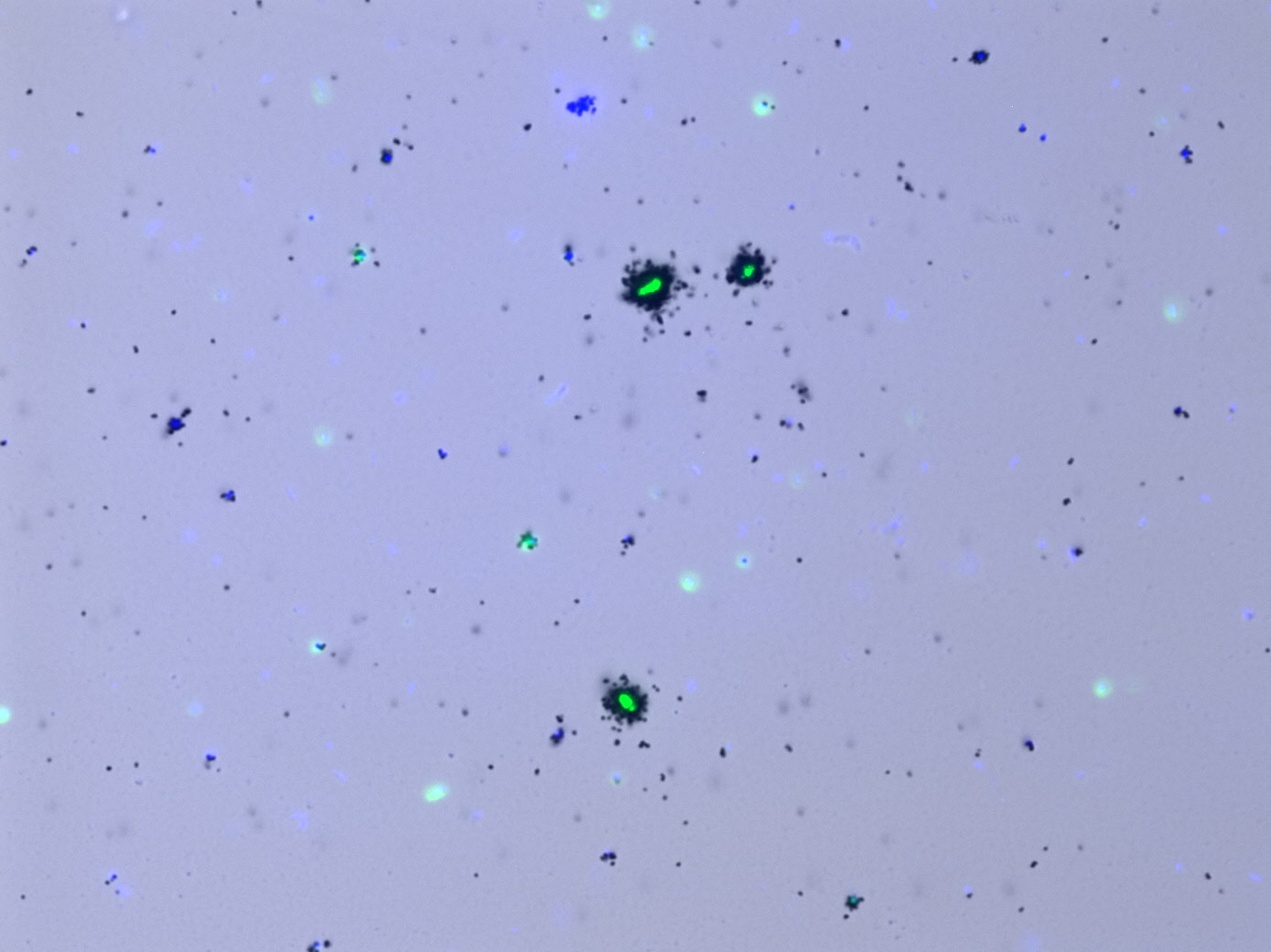

Supplement: Supplementary file 5 — Microscopic image [file 41561_2022_1081_MOESM5_ESM.jpg]
